# RESILIENT AGING ROUNDTABLE: AN EVALUATION OF A BRIEF COMMUNITY PSYCHOEDUCATION DISCUSSION GROUP

**DOI:** 10.1093/geroni/igz038.2645

**Published:** 2019-11-08

**Authors:** Andrea June, Meghan Marty

**Affiliations:** 1 Central Connecticut State University, New Britain, Connecticut, United States; 2 Rose City Geropsychology, LLC, Portland, Oregon, United States

## Abstract

Increased aging resilience levels are associated with many positive outcomes for older adults including improved quality of life, increased coping and adaptation, and decreased depressive symptoms (Earvolino-Ramirez, 2007; Fullen & Gorby, 2016; Hicks & Conner, 2014; (Sharpley, Bitsika, Wootten, & Christie, 2014). However, very few resilience promotion programs are described in the literature. The purpose of the present study was to evaluate a brief, community-based psychoeducation group designed to enhance aging resilience. Participants were recruited through a private mental health practice focused on serving older adults in the Pacific Northwest. Nine participants (M age = 71; 78% female, 100% non-Hispanic white; 100% with some college) completed the pre- and post-assessment measures: An adapted 9 item version of the Communicative Ecology Model of Successful Aging (CEMSA; Fowler, Gasiorek, & Giles, 2015) and the Groningen Ageing Resilience Inventory (GARI; van Abbema et al., 2015). The discussion group consisted of six 90-minute meetings every-other-week, facilitated by a licensed clinical psychologist. Each meeting focused on a different topic related to psycho-social aspects of aging and included understanding ageism, embracing change, creating meaning, normal and “successful” aging, and strengthening social ties. Although not statistically significant, initial results showed lower post-assessment mean scores on the CEMSA indicating lower levels of aging uncertainty, negative attributions, and pessimism as well as higher post-assessment mean scores on the GARI indicating higher perceived resilience. Moreover, 77.7 % of the group agreed or strongly agreed that they had learned a lot from the group. Future directions will be discussed.

